# Supplementary material for: Proximal renal tubular function in HIV-infected children on tenofovir disoproxil fumarate for treatment of HIV infection at two tertiary hospitals in Harare, Zimbabwe
Source: PLoS One. 2020 Jul 7;15(7):e0235759. doi: 10.1371/journal.pone.0235759 (PMC7340300; doi:10.1371/journal.pone.0235759)
Supplement: S1 File — (DOCX) [file pone.0235759.s001.docx]

**Factors associated with proteinuria in HIV infected children, on TDF for at least 6 months, < 18 years old, (n=193) ^ϯ^**

| Variable | Category | Proteinuria | | Univariate | | Multivariate**^^^** | |
| --- | --- | --- | --- | --- | --- | --- | --- |
|  |  | **Yes** | **No** | OR(95%CI) | p-value | **OR(95% CI)** | **p-value** |
| Age (years) | 0 -14 | 28 (43.1) | 63 (49.2) | 1 |  | 1 |  |
|  | ≥15 | 37 (56.7) | 65 (50.8) | 1.28 [0.70; 2.34] | 0.420 | 1.25[0.65; 2.39] | 0.505 |
|  |  |  |  |  |  |  |  |
| Gender | Female | 27 (41.5) | 60 (46.9) | 1 |  | 1 |  |
|  | Male | 38 (58.5) | 68 (53.1) | 1.24[0.68; 2.27] | 0.482 | 0.96[0.50; 1.84] | 0.899 |
|  |  |  |  |  |  |  |  |
| Current ART regimen. | TDF/3TC/EFV | 39 (60) | 96 (75) | 1 |  |  |  |
|  | TDF/3TC/NVP | 7 (10.8) | 19 (14.8) | 0.91[0.35, 2.33] | 0.839 | 0.91[0.35; 2.36] | 0.846 |
|  | TDF/3TC/PI | 19 (29.2) | 13 (10.2) | 3.60[1.62, 7.99] | **0.002*** | **3.75[1.59; 86]*** | **0.003*** |
|  |  |  |  |  |  |  |  |
| Duration on current regimen in months. | 6 -12 | 7 (10.8) | 12 (9.4) | 1 |  | 1 |  |
|  | 13 -60 | 56 (86.1) | 111 (86.7) | 0.86 [0.32; 2.32] | 0.773 | 1.25[0.42; 3.72] | 0.692 |
|  | ≥61 | 2 (3.1) | 5 (3.9) | 0.69[0.10; 4.52] | 0.695 | 1.22[0.16; 9.06] | 0.848 |
|  |  |  |  |  |  |  |  |
| Stunting | >-2SD | 48 (73.8) | 104 (81.3) | 1 |  | 1 |  |
|  | <-2SD | 11 (16.9) | 13 (10.2) | 1.83 [0.77; 4.39] | 0.173 | 1.52[0.60; 3.89] | 0.378 |
|  | <-3SD | 6 (9.2) | 11 (8.6) | 1.18 [0.41; 3.38] | 0.756 | 1.01[0.33; 3.09] | 0.987 |

^ϯ^6 samples not be processed due to a technical fault at the laboratory

*Statistically significant at α=0.05

**^** Only current ART regimen and stunting had p-value less than 0.25 in the univariate analysis. However, all variables were included in the multivariate analysis to control for any confounding effects from these variables

**Factors associated with reduction in eGFR in HIV infected children, on TDF for at least 6 months, < 18 years old, (n=190)^ϯ^**

| Variable | Category | eGFR<90ml/min/1.73m^2^ | | Univariate | | Multivariate**^^^** | |
| --- | --- | --- | --- | --- | --- | --- | --- |
|  |  | **Yes** | **No** | OR (95%CI) | p-value | OR(95% CI) | p-value |
| Age (years) | 0 -14 | 29 (42.6) | 60 (49.2) | 0.77 [0.42, 1.40] | 0.387 | 0.78[0.33; 1.84] | 0.574 |
|  | ≥ 15 | 39 (57.4) | 62 (50.8) | 1 |  | 1 |  |
|  |  |  |  |  |  |  |  |
| Gender | Female | 23 (33.8) | 64 (52.5) | 1 |  | 1 |  |
|  | Male | 45 (66.2) | 58 (47.5) | 2.16 [1.17, 3.99] | **0.014*** | 1.32[0.57; 3.08] | 0.517 |
|  |  |  |  |  |  |  |  |
| Current ART Regimen. | TDF/3TC/EFV | 38 (55.9) | 99 (81.1) | 1 |  | 1 |  |
|  | TDF/3TC/NVP | 9 (13.2) | 14 (11.5) | 1.67 [0.67, 4.19] | 0.270 | 1.05[0.29; 3.82] | 0.937 |
|  | TDF/3TC/PI | 21 (30.9) | 9 (7.4) | 6.08[2.56, 14.45] | **<0.001*** | **4.43[1.32; 4.89]*** | **0.016*** |
|  |  |  |  |  |  |  |  |
|  |  |  |  |  |  |  |  |
| Stunting | >-2SD | 47 (69.1) | 101 (82.8) | 1 |  | 1 |  |
|  | <-2SD | 11 (16.2) | 13 (10.7) | 1.82 [0.76, 4.36] | 0.180 | 1.56[0.41; 6.00] | 0.518 |
|  | <-3SD | 10 (14.7) | 8 (6.6) | 2.69 [1.00, 7.24] | **0.051** | 2.17[0.49; 9.50] | 0.305 |
|  |  |  |  |  |  |  |  |
| Baseline CD4 Count | 201 – 500 | 18 (40) | 42 (56.8) | 1 |  | 1 |  |
|  | 501 – 800 | 8 (17.8) | 16 (21.6) | 1.19 [0.43, 3.28] | 0.731 | 1.16[0.40; 3.36] | 0.788 |
|  | ≤ 200 | 11 (24.4) | 13 (17.6) | 2.02 [0.76, 5.35] | 0.156 | 1.71[0.60; 4.87] | 0.313 |
|  | ≥800 | 8 (17.8) | 3 (4) | 6.37[1.51, 6.79] | **0.012*** | **5.38[1.16; 5.04]*** | **0.032*** |

^ϯ^8 samples not processed due to a technical fault at laboratory

*Statistically significant at α=0.05

**^**Only age had a p-value greater than 0.25 in the univariate model but it was also included in the multivariate model to account for any confounding effects age may have on eGFR.
