# Supplementary material for: Proximal renal tubular function in HIV-infected children on tenofovir disoproxil fumarate for treatment of HIV infection at two tertiary hospitals in Harare, Zimbabwe
Source: PLoS One. 2020 Jul 7;15(7):e0235759. doi: 10.1371/journal.pone.0235759 (PMC7340300; doi:10.1371/journal.pone.0235759)
Supplement: S2 File — (DOCX) [file pone.0235759.s002.docx]

**Laboratory Methods**

**Urine collection**

Spot urine samples were collected during the clinic visits and immediately checked for presence of glucose and protein using the 10 parameter reagent strips (Uricheck M10, Omnipharm). Patients with positive urine glucose (>+1), had a capillary blood glucometer performed immediately (Glucoplus^TM^). The urine samples including those either glucose or protein negative dipstick were kept in a carrier cooler bag lined with ice packs between +2^0^C and +8^0^C then transported to the University of Zimbabwe, Department of Chemical Pathology laboratory within four hours of collection, for assessment of urine phosphate, creatinine and protein.

**Serum creatinine and phosphate, urine protein, phosphate and creatinine**

Blood samples for measurement of serum creatinine and phosphate were collected and transported to the laboratory in carrier bags lined with ice packs maintaining temperature between +2^0^C and +8^0^C. About 5mls of blood was collected and care was taken to avoid haemolysis. Specimens were analysed on the same day of sample collection. The Mindray BS200E Chemistry analyser manufactured by Mindray, Shenzhen, China was used to determine the concentration of creatinine, phosphate and microprotein according to the manufacturer’s instructions.

**Principle of tests**

The samples were centrifuged at 3000rpm for 5 minutes prior to testing. Urine creatinine was auto diluted 1:10 by the machine before analysis. The other laboratory test did not require dilution prior to analysis.

**Creatinine**

The analysers measures serum/urine creatinine concentration using the Modified Jaffȇ method (1, 2). In an alkaline solution, creatinine combines with picric acid to form an orange-red colored complex. The production of creatinine-picric acid complex causes an increase in absorbance at 505nm. This increase in absorbance is directly proportional to the concentration of creatinine in the sample. The equation below shows the chemical reaction scheme.

Creatinine + Picric acid OH Creatinine-Picric acid complex.

An estimation of glomerular filtration rate (eGFR) was by the Schwartz formula expressed as millimeters per minute per 1.73 meter squared (ml/min/1.73m^2^). GFR was estimated using Schwartz formula: (k*height (cm)/serum creatinine (umol/l), where k = 0.55 for children and adolescent girls and 0.77 in adolescent boys. The Schwatz formula was preferred because the modified Jaffe reaction was used. When using the modified Jaffe reaction non-creatinine chromogens are absorbed. Counham Barrat formula in which the k is reduced by 31% was preferred prior to availability of enzymatic reaction analysers. Cystatin C is even a better marker of eGFR because it is not affected by muscle mass, age, sex but the cost was prohibitive (3, 4). Renal dysfunction as indicated by reduction in glomerular filtration rate was classified as mild reduction (60-89ml/min/m^2^), moderate reduction (30-59 ml/min/m^2^), severe reduction (15-29ml/min/m^2^) and end stage renal disease (<15ml/min/m^2^) (5).

**Phosphate**

The analyser measures serum/urine phosphate concentration using the Phosphomolybdate method (6). Ammonium molybdate combines with phosphate in the presence of sulphuric acid to produce a phosphomolybdate complex. The production of phosphomolybdate complex causes an increase in absorbance at 340nm. This increase in absorbance is directly proportional to the concentration of phosphate in the sample.

The equation below shows the chemical reaction scheme.

Ammonium molybdate + Sulphuric acid + Phosphate Phosphomolybdate complex

The phosphate level was reported per age and concentration as per Division of Acquired Immunodeficiency Syndrome (DAIDS) (7) as shown in the table below

|  | Grade 1  Mild | Grade 2  Moderate | Grade 3  Severe | Grade 4 potentially life-threatening |
| --- | --- | --- | --- | --- |
| **Peadiatric >14 years** | 0.81mmol/L -<LLN | 0.65-0.80mmol/L | 0.32-0.64mmol/L | <0.32mmol/L |
| **Peadaiatric 1 year -14 years** | 0.97-1.13mmol/L | 0.81-0.96mmol/L | 0.48-0.80mmol/L | <0.48mmol/L |

**Protein**

The analyser measures urine protein concentration using the Pyrogallol red method (8). Proteins react in acid solution with pyrogallol red and molybdate to form a blue-purple colored complex. The production of the colored complex causes an increase in absorbance at 570nm. This increase in absorbance is directly proportional to the protein concentration in the sample.

The equation below shows the chemical reaction scheme.

Protien + Pirogallol red + molybdate H+ Blue-purple colored complex. Qualitative proteinuria was reported on dipstick as negative, 1+(30mg/dL), 2+ (100mg/dL), 3+(300mg/dL) or 4+(1000mg/dL) (9). Urine protein/creatinine ratio (mg/dL:mg/dL) was further performed on all urine samples. Proteinuria was defined as; normal range proteinuria if urine protein/creatinine <0.2g, further classified into intermediate proteinuria (0.2g - 3.0g) and nephrotic range proteinuria (>3.5g) (10).

**References**

1. Peake *Michael WM. Whiting M. Measurement of Serum Creatinine – Current Status and Future Goals. Clin Biochem Rev 2006; 27:173–84

2. Ou M SY, Li S, Liu G, Jia J, Zhang M, et al. LC-MS/MS Method for Serum Creatinine: Comparison with Enzymatic Method and Jaffe Method. PLOS ONE 2015; 10:e0133912

3. Berg UB NU, Bäck R, Hansson M, Monemi KÅ, Herthelius M, Björk J. New standardized cystatin C and creatinine GFR equations in children validated with inulin clearance. . Pediatr Nephrol 2015; 30:1317–26

4. Inker LA, Wyatt C, Creamer R, Hellinger J, Hotta M, Leppo M, et al. Performance of creatinine and cystatin C GFR estimating equations in an HIV-positive population on antiretrovirals. Journal of acquired immune deficiency syndromes (1999). 2012;61(3):302-9.

5. NKF KDOQI GuidelinesAccessed 15 June 2017. Available from: [<http://www2.kidney.org/professionals/kdoqi/guidelines_ckd/p5_lab_g5.htm>].

6. VK B. Serum Inorganic Phosphorus. Clinical Methods: The History, Physical, and Laboratory Examinations. Walker HK HW, Hurst JW, editors, editor. Boston: Butterworths; 1990: C. 198.

7. DAIDS adverse event clarification memo adult and pediatric adverse events grading the severity - table_for_grading_severity_of_adult_pediatric_adverse_events. 2004Accessed 3 March 2016. Available from: <http://rsc.tech-res.com/document/safetyandpharmacovigilance/table_for_grading_severity_of_adult_pediatric_adverse_events.pdf>.

8. Orsonneau JL DP, Massoubre C, Lustenberger P, Bernard S. An improved pyrogallol red-molybdate method for determining total urinary protein. Clin Chem 1989; 35:2233–6

9. Simerville JA, Maxted WC, Pahira JJ. Urinalysis: a comprehensive review. American family physician. 2005;71(6):1153-62.

10. Estrella MM, Fine DM. Screening for chronic kidney disease in HIV-infected patients. Adv Chronic Kidney Dis. 2010;17(1):26-35.
