# Supplementary material for: Proximal renal tubular function in HIV-infected children on tenofovir disoproxil fumarate for treatment of HIV infection at two tertiary hospitals in Harare, Zimbabwe
Source: PLoS One. 2020 Jul 7;15(7):e0235759. doi: 10.1371/journal.pone.0235759 (PMC7340300; doi:10.1371/journal.pone.0235759)
Supplement: S4 File — (DOCX) [file pone.0235759.s004.docx]

**DEFINITION OF TERMS**

1. **Proteinuria**

Proteinuria was defined both qualitatively and semi-quantitatively

Qualitatively: Proteinuria of ≥+1 of urine on spot dipstick urinalysis

Urine protein/creatinine ratio (mg/dL:mg/dL) (1)

1. Normal range proteinuria= urine protein/creatinine <0.2

2. Intermediate proteinuria= urine protein /creatinine ratio between 0.2g and 3.0g

3. Nephrotic range proteinuria =urine protein/ creatinine ratio >3.5g

**2.**  **Normoglycaemic glycosuria**

Normoglycaemic glycosuria was defined as > +1 glucose in spot urine dipstick and a capillary finger prick glucose < 7 mmol/L(2)

1. **Hyperphosphaturia**: Increased urinary phosphate

Hyperphosphaturia was defined as urine phosphate > 3.2mmol/dl (3)

**Estimated Glomerular Filtration Rate**

It was interpreted as:

Normal GFR > 90ml/min/1.73m^2^

Mild reduction in GFR 60-89ml/min/1.73m^2^

Moderate reduction in GFR 30-59ml/min/1.73m^2^

Severe Reduction in GFR 15-29 ml/min/1.73m^2^(4)

1. **Hypophosphatemia (Low Serum Phosphate)** (5)

|  | Grade 1  Mild | Grade 2  Moderate | Grade 3  Severe | Grade 4 potentially life-threatening |
| --- | --- | --- | --- | --- |
| **Peadiatric >14 years** | 0.81mmol/L -<LLN | 0.65-0.80mmol/L | 0.32-0.64mmol/L | <0.32mmol/L |
| **Peadaiatric 1 year -14 years** | 0.97-1.13mmol/L | 0.81-0.96mmol/L | 0.48-0.80mmol/L | <0.48mmol/L |

1. **WHO Anthropometric classification for children and adolescents 5-19 years**

**WHO Classification of children and adolescents 5-19 years of age BMI-for-age Z-score(6)**

| Severe Thinness | <-3SD |
| --- | --- |
| Thinness | ≥ -3 SD & < -2 SD |
| Overweight | >+1SD & ≤+2 SD (equivalent to BMI 25 kg/m2 at 19 years) |
| Obesity | >+2SD (equivalent to BMI 30 kg/m2 at 19 years) |

**WHO classification of children and adolescents 5-19 year’s height for age Z score(7)**

| Severe stunting | <-3SD |
| --- | --- |
| Moderate stunting | <-2SD |
| Normal | >-2SD |

1. Simerville JA, Maxted WC, Pahira JJ. Urinalysis: a comprehensive review. American family physician. 2005;71(6):1153-62.

2. Version MMC. Renal Glucosuria - Kidney and Urinary Tract Disorders Internet [cited 2017 Jun 12] Available from: <http://wwwmerckmanualscom/home/kidney-and-urinary-tract-disorders/disorders-of-kidney-tubules/renal-glucosuria>.

3. Bansal VK. Serum Inorganic Phosphorus. In: rd, Walker HK, Hall WD, Hurst JW, editors. Clinical Methods: The History, Physical, and Laboratory Examinations. Boston: Butterworths

Butterworth Publishers, a division of Reed Publishing.; 1990.

4. Levey AS, Eckardt K-U, Tsukamoto Y, Levin A, Coresh J, Rossert J, et al. Definition and classification of chronic kidney disease: A position statement from Kidney Disease: Improving Global Outcomes (KDIGO). Kidney International. 2005;67(6):2089-100.

5. DAIDS adverse event clarification memo adult and pediatric adverse events grading the severity - table_for_grading_severity_of_adult_pediatric_adverse_events. 2004Accessed 3 March 2016. Available from: <http://rsc.tech-res.com/document/safetyandpharmacovigilance/table_for_grading_severity_of_adult_pediatric_adverse_events.pdf>.

6. WHO. BMI classification tables for children 5-19 years to BMI who - Google Search. Internet[cited 2017 Feb 26] Available from: <https://wwwgooglecom/search?q=Table++The+International+Classification+of++5-19+yeaarsadolescents++underweight%2C+overweight+and+obesity+according+to+BMI&ie=utf-8&oe=utf-8#safe=active&q=BMI+classification+tables+for+children+5-19+years+++to+BMI+who>. 2017.

7. WHO. Height-for-age (5-19 years) [Internet]. WHO. [cited 2017 Jun 12] Available from: <http://wwwwhoint/growthref/who2007_height_for_age/en/>.
