# Supplementary material for: Proximal renal tubular function in HIV-infected children on tenofovir disoproxil fumarate for treatment of HIV infection at two tertiary hospitals in Harare, Zimbabwe
Source: PLoS One. 2020 Jul 7;15(7):e0235759. doi: 10.1371/journal.pone.0235759 (PMC7340300; doi:10.1371/journal.pone.0235759)
Supplement: Attachment — Submitted filename: Rebuttal letter PLOS ONE comments.docx [file pone.0235759.s005.docx]

14 May 2020

Dear Editor

Firstly, we would like to thank The PLOS ONE for considering our manuscript ‘’PONE-D-19-5784: Proximal renal tubular function in HIV-infected children on tenofovir disoproxil fumarate for treatment of HIV infection at two tertiary hospitals in Harare, Zimbabwe’’ for review. Secondly, we appreciate the reviewers’ and Editor’s comments. The authors responded to comments and the authors’ changes are highlighted in the main manuscript labeled ‘Manuscript with track changes’. Below are the responses for the two reviewers.

**Reviewers’ comments**: To enhance the reproducibility of your results, we recommend that if applicable you deposit your laboratory protocols in protocols.io, where a protocol can be assigned its own identifier (DOI) such that it can be cited independently in the future. For instructions see: <http://journals.plos.org/plosone/s/submission-guidelines#loc-laboratory-protocols>

**Authors’ Response**: Thank you for the suggestion. The laboratory protocol was deposited in protocols.io and can be found on the following link: **protocols.io**<https://protocols.io/view/pone-d-19-35784-proximal-renal-tubular-function-in-bfzxjp7n> and [dx.doi.org/10.17504/protocols.io.bfzxjp7n](https://dx.doi.org/10.17504/protocols.io.bfzxjp7n). The link is included in the manuscript under the methodology section page 7, line 154 -155.

**Reviewer’s comments:** The PLOS Data policy requires authors to make all data underlying the findings described in their manuscript fully available without restriction, with rare exception (please refer to the Data Availability Statement in the manuscript PDF file). The data should be provided as part of the manuscript or its supporting information or deposited to a public repository. For example, in addition to summary statistics, the data points behind means, medians and variance measures should be available. If there are restrictions on publicly sharing data—e.g. participant privacy or use of data from a third party—those must be specified.

**Authors’ response:** The authors excluded any data that would identify participants. Supporting information with data tables and MDB file were uploaded: DOI https://doi.org/10.5061/dryad.2fqz612mf. For private access prior to publication use <https://datadryad.org/stash/share/pRmK7SDWzk5Jnd3-usw2hKCGVe71OksxVKbxfTZv5PE>. The link is included in the main manuscript page 8 line 165-166.

**Reviewer #1 Comments**: Even though the sample size is not so big, the study is well presented and gives relevant data in the use of TDF. In the methods they recruited children that were on that regimen for at least six months, but it would have been interesting to check the differences depending on how long they were on the treatment. The conclusions are quite well presented and they also describe properly the most important limitations that it is that they did not compare a groups with other regimens.

In conclusion, the article is technically sound and gives the data needed for the conclusions and the author’s know and stand clear the limitations of the study.

**Authors’ comments**: Thank you for the comments. A comparison of effect of TDF depending on how long participants were on the treatment is discussed on the section on glomerular function and proteinuria. Figure 1 and 2 summarizes the finding based on duration on treatment.

**Reviewer #2**: Dr Mashingaidze-Mano and colleagues studied 198 children on TDF-based ARV therapy. This is a fantastic report and important contribution to the literature. The finding that 1/3 had abnormal proteinuria is extremely important and appears to be downplayed somewhat in the authors conclusions. The authors conclude a “low prevalence of proximal tubular dysfunction”. Proteinuria (particularly non-albumin proteinuria that typifies TDF toxicity) is a marker of tubular dysfunction.

Most importantly: I believe proteinuria (when confirmed by UPC ratio) should be included as a tubular abnormality and the entire prevalence of “tubular dysfunction” recalculated.

**Authors’ response**: Thank you for the comments and suggestions. Urine albumin/total protein ratio (uAPR) < 0.4 identifies tubular pathology in proteinuric patients and this was not measured in our study (1). Hence, proteinuria was not included as a marker of tubular function as we had not separated the two. This can however be included in the future studies.

**Reviewer’s comment**: The intro is too long and the information about genetic deteminants (ABCC2 ) should be moved to discussion

**Authors’ Response**: Thank you for the observation. The introduction was shortened and the information about genetic determinants (ABCC2 ) was moved to discussion section.

**Reviewer’s comment**: Severe stunting should be defined.

**Authors’ comments**: Severe stunting was defined as Height for age z score < -3standard deviation as per the World Health Organization nutritional status classification. This definition has been added on page 6 line 125 to 126 of the main manuscript the authors had indicated that: The WHO growth charts (2007) were used to determine nutritional status of the study participants (2). An additional references to define stunting and body mass index were added as well as (3, 4).

**Reviewer’s comment**: Define proteinuria cutoffs used for UPC ratios

**Authors’ response**: Thank for highlighting such an important omission, which has now been added from **line 131 to 135 on page** 6. Qualitative proteinuria was reported on dipstick as negative, 1+ (30mg/dL), 2+ (100mg/dL), 3+ (300mg/dL) or 4+(1000mg/dL). Urine protein/creatinine ratio (mg/dL:mg/dL) was further performed on all urine samples (5). Proteinuria was defined as; normal range proteinuria if urine protein/creatinine <0.2g, further classified into intermediate proteinuria (0.2g - 3.0g) and nephrotic range proteinuria (>3.5g) (6).

**Reviewer’s comments**: Spell out DAIDS on first use

**Authors: Response**: DAIDS has now been spelt out as: “Division of Acquired Immunodeficiency Syndrome ’’ (7). The full definition and grading is within document definition of terms S5 supplementary information.

**Reviewers’ Comments:** This article is generally well written but the discussion is confusing when discussing predictors of proteinuria. This should be reworded: “Longer duration on TDF was not a good predictor of detection of proteinuria. In this study, the odds of having proteinuria in patients on TDF for >60 months was 0.69 CI (0.10; 4.52) compared to those with shorter duration.” Do you mean “negative predictor” since the OR was < 1?? The following sentence is also confusing: “Exposure to PI regimen was a good predictor for having proteinuria.” This is not conventional language for discussing predictors.

**Authors’ Response:** The authors are grateful for the comments. The sentences Longer duration on TDF was not a good predictor of detection of proteinuria. In this study, the odds of having proteinuria in patients on TDF for >60 months was 0.69 CI (0.10; 4.52) compared to those with shorter duration.” has been reworded to Longer duration of TDF was a negative predictor of proteinuria. In this study, the odds of having proteinuria in patients on TDF for >60 months was 0.69 CI (0.10; 4.52) compared to those with shorter duration. The following sentences has also been reworded from Exposure to PI regimen was a good predictor for having proteinuria.” Exposure to PI was a positive predictor of having proteinuria. **See page 16 line 328 and 333**

**Reviewers Comment:** TAF should be spelled out in first use and authors should explain differences from TAF to TDF, explain to reader why less nephrotoxic

**Authors’ Response**: Thank you for the observation. A statement has added to explain why (tenofovir alafenamide (TAF) is less nephrotoxic: TAF's unique pharmacokinetic profile enables provision of lower required doses for antiviral efficacy. Lower concentrations reach renal tubules minimizing intracellular accumulation and mitochondrial damage hence less nephrotoxicity compared to TDF (8)

**References**

1.	Samarawickrama A, Cai M, Smith E, Nambiar K, Sabin C, Fisher M, et al. Simultaneous measurement of urinary albumin and total protein may facilitate decision-making in HIV-infected patients with proteinuria. HIV Medicine. 2012;13(9):526-32.

2.	WHO. The WHO Child Growth Standards. 2009.

3.	WHO. Height-for-age (5-19 years) [Internet]. WHO. [cited 2017 Jun 12] Available from: <http://wwwwhoint/growthref/who2007_height_for_age/en/>.

4.	WHO.	BMI classification tables for children 5-19 years to BMI who - Google Search. Internet[cited 2017 Feb 26] Available from: <https://wwwgooglecom/search?q=Table++The+International+Classification+of++5-19+yeaarsadolescents++underweight%2C+overweight+and+obesity+according+to+BMI&ie=utf-8&oe=utf-8#safe=active&q=BMI+classification+tables+for+children+5-19+years+++to+BMI+who>. 2017.

5.	Simerville JA, Maxted WC, Pahira JJ. Urinalysis: a comprehensive review. American family physician. 2005;71(6):1153-62.

6.	JT D. Handbook of Chronic Kidney Disease Management. . 2012:656.

7.	DAIDS adverse event clarification memo adult and pediatric adverse events grading the severity - table_for_grading_severity_of_adult_pediatric_adverse_events. 2004Accessed 3 March 2016. Available from: <http://rsc.tech-res.com/document/safetyandpharmacovigilance/table_for_grading_severity_of_adult_pediatric_adverse_events.pdf>.

8.	Novick TK, Choi MJ, Rosenberg AZ, McMahon BA, Fine D, Atta MG. Tenofovir alafenamide nephrotoxicity in an HIV-positive patient: A case report. Medicine (Baltimore). 2017;96(36):e8046-e.
